# Impact of Fentanyl on Recovery Times and Hypotensive Events in Ophthalmic Surgery: A Comparative Study

**DOI:** 10.3390/medicina61020282

**Published:** 2025-02-06

**Authors:** Garegin Manukyan, Daniel Beel, Marcus Thudium, Christina Katharina Weisheit

**Affiliations:** 1 Department of Anesthesiology and Intensive Care Medicine, University Hospital Bonn, 53127 Bonn, Germany; garegin.manukyan@ukbonn.de (G.M.); daniel.beel@ukbonn.de (D.B.); marcus.thudium@ukbonn.de (M.T.); 2 Department of Anesthesiology and Intensive Care Medicine, Faculty of Medicine, University Hospital Cologne, University of Cologne, 50937 Cologne, Germany

**Keywords:** fentanyl, remifentanil, ophthalmology, discharge from recovery ward, vasopressor consumption

## Abstract

*Background and Objectives*: Remifentanil is a member of the fentanyl family and a short-acting, esterase-metabolized opioid that offers potential advantages over fentanyl in ophthalmic surgeries characterized by rapid patient turnover. This study aimed to compare two different analgesia induction regimes, remifentanil or fentanyl, with respect to intraoperative hypotensive events and perioperative process times in patients undergoing ophthalmic surgery under general anesthesia. *Materials and Methods*: Clinical data of 500 patients either receiving remifentanil infusion (R group, 0.4–0.5 μg/kg/min at induction, and then 0.1 µg/kg/min maintenance dose) or fentanyl bolus (F group, 1 μg/kg for induction followed by 0.1 μg/kg/min remifentanil maintenance dose) were analyzed in this retrospective study. All patients received a propofol injection as part of the induction and sevoflurane for the maintenance of anesthesia. We investigated hemodynamic events as defined by the administration of vasopressors, atropine and Akrinor (Theodrenaline and Cafedrine), as well as procedural times in the two groups. *Results*: There was no difference in hypotensive events between the two groups. However, there was a relationship between preoperative ASA (American Society of Anesthesiologists) status and vasopressor administration. The amount of propofol for the induction of anesthesia exhibited a significant correlation to the age of the patients (*p* < 0.05). The time from the end of anesthesia to discharge from the recovery room was significantly reduced by approximately 6 min per patient in the remifentanil group (*p* < 0.01). In conclusion, the induction of anesthesia with high-dose remifentanil combined with propofol can significantly shorten the time span to discharge from the recovery ward compared to fentanyl bolus administration, without an increase in the administration of vasopressors in patients undergoing ophthalmic surgery in general anesthesia. *Conclusions*: These findings suggest that remifentanil may be a more effective choice for anesthesia management in settings with high patient turnover.

## 1. Introduction

Ophthalmic surgery places special requirements on anesthesia. Surgical interventions in ophthalmology are very short compared to other surgical disciplines, with an average duration of 50 min (own data), and fast patient turnover characterizes good management. On the other hand, deep anesthesia is needed to ensure reliable akinesia and analgesia. Gild et al. reported that sudden movements or coughing were the cause of injury in 30% of cases with legal consequences for the anesthesiologist [1]. The demands can be met by the high-dose administration of opioids, muscle relaxants and volatile anesthetics. However, the administration of large amounts of opioids harbors the risk of opioid surplus, potentially resulting in increased recovery times with longer postoperative monitoring. This kind of prolonged stay in the recovery room with monitoring would generally not be necessary concerning the surgical intervention.

The opioid remifentanil offers a way out of this dilemma as large doses can be administered, which assures deep intra-operative analgesia and while permitting rapid extubation and awakening at the end of the procedure. Due to the unique pharmacodynamics of remifentanil, the context-sensitive half-time does not increase with the duration of drug administration. Therefore, the patient can be quickly extubated and prolonged monitoring and recovery are not required [2]. The potential disadvantage is the rapid loss of effectiveness and the occurrence of postoperative pain which can be considered minimal in ophthalmic surgery as intraoperative topical and regional anesthesia reduce postoperative pain [2]. Remifentanil, unlike fentanyl, is metabolized by non-specific blood and tissue esterase’s, resulting in a context-insensitive half-life that remains constant regardless of infusion duration. This pharmacokinetic profile enables precise titration and rapid recovery after discontinuation, making it particularly well-suited for short procedures. Moreover, Remifentanil’s clearance is predictable and unaffected by organ dysfunction, which is especially advantageous in elderly or high-risk populations [3].

In contrast, fentanyl has a longer and context-sensitive half-life due to its redistribution and hepatic metabolism. This can lead to prolonged postoperative sedation and necessitate extended monitoring, especially in procedures requiring repeated bolus administration or prolonged infusions [4].

Remifentanil is special compared to all other opioids as it possesses an ester linkage that ensures that the pharmacokinetics are predictable and makes it particularly useful when rapid onset and offset of opioid effects are desirable [5], as it is typically the case in ophthalmic surgery.

Despite the higher costs, the additional cost of remifentanil may be justified by improved outcomes or reduced recovery time [4].

In a retrospective study, we analyzed the data of 500 ophthalmic surgery patients with general anesthesia to uncover the clinical and process time effects of using remifentanil in comparison to fentanyl for anesthesia induction.

We hypothesized the effect of the opioid regimen on intraoperative hypotensive events and bradycardia as well as on perioperative process times.

## 2. Materials and Methods

### 2.1. Study Design

The study was conducted in accordance with good clinical practice and the Declaration of Helsinki. After approval from the local ethics committees, a total of 500 patients were included. In a retrospective chart review, digital anesthesia protocols were evaluated and 250 patients per group were screened, receiving either a remifentanil–propofol- or fentanyl–propofol-based anesthesia induction.

### 2.2. Statistics

Statistical analyses were conducted using R and R Studio. Continuous variables were tested for normality using the Shapiro–Wilk test. Since some data did not follow a normal distribution, non-parametric tests were employed. Specifically, the Wilcoxon rank-sum test was used to compare continuous variables, such as anesthesia time, surgery time and cumulative doses of administered drugs between the remifentanil and mixed opioid groups. To quantify the effect of our results we calculated the effect size ‘r’. Results are presented as median values with interquartile ranges (IQR) or as means with standard deviations (SD) where appropriate.

For categorical variables, such as the incidence of hypotensive and bradycardic events, as well as the administration of vasopressors (e.g., norepinephrine and Akrinor) and atropine, Pearson’s Chi-squared test with Yates’ continuity correction was applied to assess differences between the groups. The significance of the association between these variables and potential confounders, including age, ASA classification and opioid regimen, was evaluated using logistic regression analysis. Odds ratios (ORs) with 95% confidence intervals (CIs) were calculated to quantify the strength of associations.

Interaction terms were considered in the models to explore potential effect modifications by age and ASA status on the relationship between opioid use and perioperative outcomes. Statistical significance was defined as a *p*-value of less than 0.05. All analyses were two-tailed. Data management and statistical analyses were performed in accordance with the principles of good clinical practice, ensuring the integrity and reproducibility of the results.

## 3. Results

### 3.1. Patient Demographics and Clinical Characteristics

**Figure 1:**
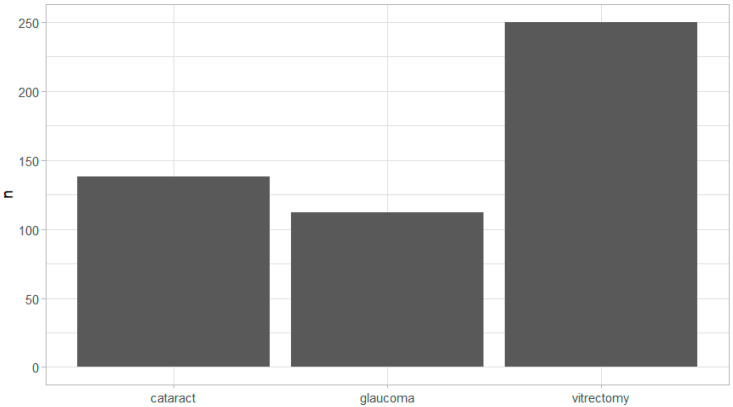
The distribution of surgeries for all included patients of both the R and F group (*n* = 500) indicates the number of patients. Data show that the majority of patients (50%) underwent vitrectomy.

A total of 500 patients were included in this retrospective study. Demographic and clinical characteristics are summarized in Table 1. Half of the patients were in the remifentanil (R) group and the other half in the fentanyl (F) group. The average duration of surgery time was 50 min, ventilation time 61 min and total anesthesia time 83 min. The patients included underwent vitrectomy, cataract or glaucoma surgery (Figure 1).

### 3.2. Procedural Times

The mean surgery time was 50.18 min (SD ± 24.27) for the entire dataset. For the combined use of remifentanil and fentanyl, the mean was 50.74 min (SD ± 23.67), while for remifentanil only, it was 49.64 min (SD ± 24.89). The difference was not statistically significant (*p* = 0.49). The ventilation time averaged 60.94 min (SD ± 24.19) across all patients, with 61.33 min (SD ± 24.31) for the combined opioid group and 60.54 min (SD ± 24.12) for the remifentanil-only group. This difference was also not statistically significant (*p* = 0.81).

The duration of anesthesia time, defined as the time from first contact with the anesthesia team to discharge from the post-anesthesia care area, was 82.97 min (SD ± 28.9) for the whole dataset. In the combined group, it was 86.4 min (SD ± 29.2), while the remifentanil-only group had a mean of 79.55 min (SD ± 28.24). The difference reached statistical significance (*p* = 0.03). Investigating the duration of anesthesia time, defined as the time between first contact with the anesthesia team and discharge from the post-anesthesia care area, we found a duration of 82.97 min (SD ± 28.9 m) for the whole dataset, 86.4 min (SD ± 29.2 m) for the combined use of remifentanil and fentanyl and 79.55 min (SD ± 28.24) for remifentanil only. In the remifentanil-only group, the mean anesthesia time was about six minutes shorter than in the combined group and reached statistical significance (*p* = 0.03).

### 3.3. Hemodynamic Events

We evaluated whether the choice of opioid regimen influenced adverse hemodynamic events such as hypotension or bradycardia, as well as the duration of surgery, ventilation time or total anesthesia time.

Hypotension, defined by the administration of norepinephrine or Akrinor, occurred in 239 (95.6%) patients receiving remifentanil only and 245 (98%) patients receiving both remifentanil and fentanyl. A chi-squared test revealed no significant difference in hypotension incidence between the two groups (chi-square = 1.6142, *p* = 0.20).

Bradycardia, indicated by atropine administration, was observed in 73 (29.2%) patients receiving remifentanil only and 77 (30.8%) patients receiving both opioids. Again, no significant difference was found (chi-square = 0.0857, *p* = 0.76). The opioid regimen did not influence the duration of surgery or ventilation time in our study.

### 3.4. Vasopressor Requirements

We found no significant difference in the cumulative dose of Akrinor or the maximum dose of norepinephrine based on the opioid regimen. The remifentanil plus fentanyl group received a mean of 0.02 (SD ± 0.1) units of Akrinor, while the remifentanil-only group received a mean of 0.04 units (*p* = 0.881).

The maximum norepinephrine administered was slightly lower in the remifentanil-only group (5.09 µg/min, SD ± 2.91) compared to the combined group (5.21 µg/min, SD ± 3.43), but this difference was not statistically significant (*p* = 0.882).

### 3.5. Influence of Age and Comorbidities

**Figure 2:**
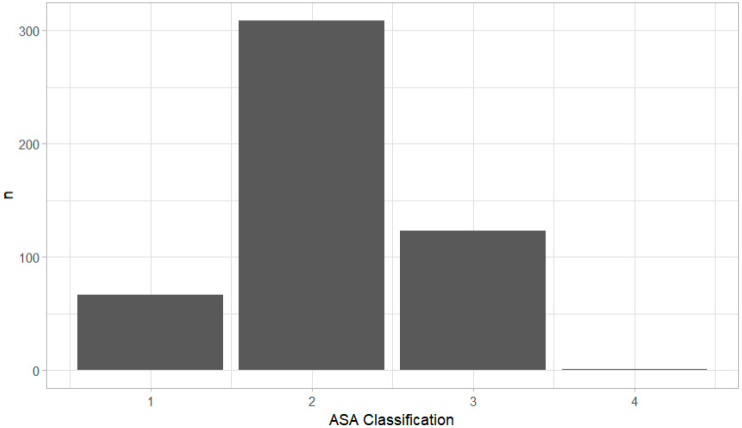
Distribution of preoperative ASA status according to the updated ASA classification for all patients. Data show that the majority of patients is in the ASA II or III group. ASA I: *n* = 67; ASA II: *n* = 308; ASA III: *n* = 123; ASA IV: *n* = 1; ASA: American Society of Anesthesiologists.

The majority of patients were pre-operatively classified as ASA II (Figure 2).

While a correlation was noted between patient age and ASA classification, age did not influence total anesthesia time, operation time or ventilation time. Older patients received higher levels of remifentanil monotreatment and less propofol compared to younger patients. Notably, older patients exhibited significantly higher norepinephrine consumption, with no difference observed in atropine and Akrinor usage.

In terms of vasopressor administration, significantly more ASA II patients received vasopressors compared to ASA I patients (88% vs. 98%, *p* < 0.001), and similarly when comparing ASA I to ASA III patients (88% vs. 99%, *p* < 0.001). All patients older than 65 years experienced hypotension, while 93% of patients aged 65 years or younger required vasopressors (*p* < 0.001). The incidences of hypotension according to ASA classification are provided in Supplementary Materials.

### 3.6. Propofol Administration

A slight difference was noted in the amount of propofol used for anesthesia induction. Patients receiving the combined analgesia of remifentanil and fentanyl required a mean of 202.52 mg (SD ± 98.46), while those on remifentanil only received a mean of 196.61 mg (SD ± 116.9). This difference reached statistical significance (*p* = 0.016). These findings and their clinical implications will be discussed further in this paper.

## 4. Discussion

In our retrospective analysis of 500 patients receiving either remifentanil infusion (R group) or fentanyl bolus (F group), we found no difference between the groups concerning hypotensive events or bradycardia requiring interventions. Previous studies have suggested an association of remifentanil with a higher rate of intraoperative hypotension and muscle rigidity compared to fentanyl [5,6]. Our findings contradict the results of a recently published study by Girish et al. that indicated that remifentanil was associated with more intraoperative hypotension than fentanyl [6]. However, this study could not highlight any significant differences between the two drugs with respect to other adverse events (i.e., episodes of hypertension, bradycardia, respiratory depression and apnea) which is consistent with our results.

There are several reasons for the absence of a hypotensive effect in the remifentanil group in our study. It was our hypothesis that in the F group, the combination of opioids may lead to hypotension or bradycardia. Since this is a retrospective analysis and opioid doses were administered according to the request of the anesthesiologist and with regard to minimal trauma in ophthalmological surgery one could also suspect that overdosing opioids in the R group did not. Another possibility is that transient and discrete hypotensive events were missed by the monitoring used in our setting. Since non-continuous blood pressure measurement was used, hypotension could take place in the time between the measurement interval which is set to 2.5 min by standard. Although it seems unlikely, such transient hypotensive episodes may influence the results presented here, but they may also represent a significant safety issue in patients at risk. Wijnberge et al. reported that 92% of hypotensive events were detected with non-continuous blood pressure measurements, but with a delay of up to two minutes. Incidences of hypotension of 77% to 84% have been reported previously [7] compared to 97% in our patients. This may lead to the conclusion that continuous blood pressure monitoring even with non-invasive methods and guidance with machine learning algorithms may improve the identification of hypotensive events.

Irrespective of the choice of opioids applied, we found that increased vasopressor administration correlated with patient age. All patients above the age of 65 received vasopressors during anesthesia. This underlines the importance of adequate monitoring and vigilance of the anesthesiologists to avoid hypotensive events which may potentially result in complications.

Even in the age group < 65 years, vasopressors were used in most patients, suggesting that even in a low-impact setting as ophthalmic surgery, treating for hypotension should be considered the rule rather than the exception and especially points to the high vulnerability of older patients in this setting. The recently published consensus statement on perioperative arterial pressure management highlights that intraoperative mean arterial pressures < 60–70 mm Hg or systolic arterial pressures < 90–100 mm Hg are associated with acute kidney injury, myocardial injury, myocardial infarction and death; intraoperative mean arterial pressure should be maintained at ≥60 mm Hg in at-risk patients [8].

### 4.1. Implications of Faster Recovery Times with Remifentanil

One of the notable findings from our study is the reduced anesthesia time associated with remifentanil compared to fentanyl, with a significant difference of approximately 6 min (*p* = 0.03). While this may seem minor, in a high-turnover surgical environment, such as ophthalmic surgery, this reduction can lead to substantial time savings. For instance, with a daily caseload of 10–12 patients, this could translate to over 60 min of excess anesthesia time per day, effectively equating to one additional surgical procedure. Our findings are supported by a recently published study of Lin et al. concluding that remifentanil resulted in faster recovery than alfentanil in endoscopic ultrasound-guided tissue acquisition [8]. Therefore, our results suggest that there is no rationale for using fentanyl in a setting as described here.

The pharmacokinetic properties of remifentanil, which allow for rapid onset and offset of action, could make it a more suitable choice in settings where patient turnover is critical. The potential for quicker recovery times not only enhances operational efficiency but may also improve patient satisfaction and decrease the risk of complications associated with prolonged anesthesia. This is particularly relevant for elderly or obese patients, who may be more vulnerable to the effects of longer anesthesia durations. Additionally, the possibility of desaturation events on the normal ward has to be kept in mind, which are more likely when fentanyl is used. Therefore, according to our results, the use of fentanyl for anesthesia induction cannot be recommended in an ophthalmic surgical setting.

### 4.2. Comparison with Previous Studies on Patient Safety and Efficiency

Our findings align with the growing body of literature suggesting that remifentanil may offer advantages in terms of recovery times without compromising safety. Although the incidence of hypotension was high across both groups, particularly in patients over 65 years, the absence of a significant difference in hypotensive events between the two opioid regimens suggests that the choice of opioid may not be the sole determinant of hemodynamic stability. The absence of significant hypotensive events in our study further supports the notion that remifentanil may provide a safer alternative in surgical settings.

Moreover, the increased vasopressor administration correlated with patient age underscores the necessity for vigilant monitoring by anesthesiologists to mitigate hypotensive events, especially in older patients. The consensus statement on perioperative arterial pressure management emphasizes that maintaining intraoperative mean arterial pressures ≥ 60 mm Hg is crucial to prevent complications such as acute kidney injury and myocardial events [8].

Our data revealed that patients in the R group received less Propofol compared to the patients in the F group for the induction of general anesthesia. This finding is reflected by a study of males that indicates an association between the use of remifentanil and a reduction in propofol dose [9]. The colleagues state that in their setting, remifentanil has larger drug acquisition costs but does not increase the total hospital costs associated with cardiac surgery. While our study did not find a direct effect of opioid regimen on hypotensive events, the data highlights the importance of considering recovery times and the implications for procedural efficiency. The increased recovery time associated with fentanyl raises questions about its utility in high-turnover settings, suggesting that remifentanil may provide a safer and more efficient alternative.

In summary, from our results we can deduce the following recommendations for clinical practice: Remifentanil should be used instead of fentanyl for anesthesia induction in high-turnover settings to reduce recovery times. Furthermore, hemodynamic events are common even in low-impact surgeries irrespective of the opioid regimen and require adequate therapy. This is especially in patients with higher ASA status.

## 5. Conclusions

While we did not observe significant differences in hemodynamic events between the two opioid regimens, the high incidence of hypotension, particularly in patients over 65 years, cannot be overlooked. The association of fentanyl with increased recovery times suggests that remifentanil may be more appropriate for use in settings with high patient turnover, particularly in ophthalmic surgery. Future studies should continue to explore the balance between opioid choice, recovery efficiency and patient safety to optimize anesthetic practices.

## Figures and Tables

**Table 1:** Patient demographics and clinical characteristics. Data are indicated for all patients (*n* = 500), the F group (fentanyl for induction, remifentanil for maintenance, *n* = 250) and R group (remifentanil for induction and maintenance, *n* = 250). Data given as mean ± standard deviation.

	All Patients	Remifentanil + Fentanyl	Remifentanil	*p*	r
Age [y]	65.76 ± 13.53	66.16 ± 13.48	65.36 ± 13.6	0.428	
Weight [kg]	79.41 ± 17.92	78.5 ± 17.1	80.32 ± 18.68	0.489	
Gender Female [*n*]	238	118	120		
Gender Male [*n*]	262	130	132		
ASA I [*n*]	67	37	30		
ASA II [*n*]	309	152	157		
ASA III [*n*]	123	61	62		
ASA IV [*n*]	1	0	1		
Anesthesia time [min]	82.97 ± 28.9	86.4 ± 29.2	79.55 ± 28.24	0.003	0.13
Surgery time [min]	50.18 ± 24.27	50.74 ± 23.67	49.64 ± 24.89	0.448	
Ventilation time [min]	60.94 ± 24.19	61.33 ± 24.31	60.54 ± 24.12	0.812	
Remifentanil CD [µg]	478.30 ± 259.43	418.59 ± 234.26	538.02 ± 269.89	<0.001	0.26
Fentanyl CD [µg]	0.05 ± 0.05	0.10 ± 0.01	0		
Propofol CD [mg]	199.57 ± 108	202.52 ± 98.46	196.61 ± 116.9	0.016	0.19
Akrinor CD [amp]	0.03 ± 0.11	0.02 ± 0.1	0.04 ± 0.13	0.881	
Norepinephrine MD [µg/min]	5.15 ± 3.18	5.21 ± 3.43	5.09 ± 2.91	0.882	
Cristalloids [ml]	745 ± 278.62	766 ± 273.94	724 ± 282.21	0.053	

CD = cumulative dose; MD = maximum dose; r = effect size.

## Data Availability

All relevant data are in the manuscript.

## Supplementary Materials

The following supporting information can be downloaded at: https://www.mdpi.com/article/10.3390/medicina61020282/s1, Supplemental Table S1: ASA and patient age vs hypotensive event.

## Abbreviations

The following abbreviations are used in this manuscript:

CD	Cumulative Dose
MD	Maximum Dose

## References

[1] Gild W.M., Posner K.L., Caplan R.A., Cheney F.W. (1992). Eye injuries associated with anesthesia. A closed claims analysis. Anesthesiology.

[2] Komatsu R., Turan A.M., Orhan-Sungur M., McGuire J., Radke O.C., Apfel C.C. (2007). Remifentanil for general anaesthesia: A systematic review. Anaesthesia.

[3] Muellejans B., López A., Cross M.H., Bonome C., Morrison L., Kirkham A.J. (2004). Remifentanil versus fentanyl for analgesia based sedation to provide patient comfort in the intensive care unit: A randomized, double-blind controlled trial [ISRCTN43755713]. Crit. Care.

[4] Beers R.A., Calimlim J.R., Uddoh E., Esposito B.F., Camporesi E.M. (2000). A comparison of the cost-effectiveness of remifentanil versus fentanyl as an adjuvant to general anesthesia for outpatient gynecologic surgery. Anesth. Analg..

[5] Rosow C.E. (1999). An overview of remifentanil. Anesth. Analg..

[6] Joshi G.P., Warner D.S., Twersky R.S., Fleisher L.A. (2002). A comparison of the remifentanil and fentanyl adverse effect profile in a multicenter phase IV study. J. Clin. Anesth..

[7] Wijnberge M., van der Ster B., Vlaar A.P.J., Hollmann M.W., Geerts B.F., Veelo D.P. (2022). The Effect of Intermittent versus Continuous Non-Invasive Blood Pressure Monitoring on the Detection of Intraoperative Hypotension, a Sub-Study. J. Clin. Med..

[8] Lin Y.J., Wang Y.C., Huang H.H., Huang C.H., Lin P.L. (2022). Efficacy and safety of remifentanil for endoscopic ultrasound-guided tissue acquisition: A single center retrospective study. Surg. Endosc..

[9] Myles P.S., Hunt J.O., Fletcher H., Watts J., Bain D., Silvers A., Buckland M.R. (2002). Remifentanil, fentanyl, and cardiac surgery: A double-blinded, randomized, controlled trial of costs and outcomes. Anesth. Analg..

