# Supplementary material for: Impact of Fentanyl on Recovery Times and Hypotensive Events in Ophthalmic Surgery: A Comparative Study
Source: Medicina (Kaunas). 2025 Feb 6;61(2):282. doi: 10.3390/medicina61020282 (PMC11857306; doi:10.3390/medicina61020282)
Supplement: Supplementary file 1 [file medicina-61-00282-s001.zip › medicina-3409677-supplementary.pdf]

parameter	no hypotension	hypotension	p	odds ratio
**ASA**				
**I**	8	59	-	-
**II**	7	302	<0.001	1.77
**III**	1	122	<0.002	2.81
**IV**	0	1	1	-
**Age, years**				
**≤65**	16	279	-	-
**>65**	0	205	<0.001	3.80

Supplemental Table S1: ASA and patient age vs hypotensive event. Hypotensive events were defined as the need of norepinephrine or Akrinor administration. Comparisons in ASA groups were performed in relation to ASA I patients.
